# Developing the next generation of medical educators: assessing the impact of a clinical teaching elective offered to senior medical students

**DOI:** 10.5116/ijme.6209.14d0

**Published:** 2022-03-07

**Authors:** Karima Khamisa, Ilan A. Fellus, Olga O. Fellus, Warren J. Cheung, Melissa Rousseau

**Affiliations:** 1 Division of Hematology, Department of Medicine, Ottawa Blood Diseases Centre, The Ottawa Hospital, Canada; 2 Emergency Department, Markham Stouffville Hospital, Canada; 3 Faculty of Education, University of Ottawa, Canada; 4 Department of Emergency Medicine, Faculty of Medicine, University of Ottawa, Canada; 5 Division of General Internal Medicine, Department of Medicine, Canada

**Keywords:** Curriculum development, undergraduate medical education, teaching skills, culture of teaching, students as teachers

## To the Editor

Teaching junior trainees and peers is a core skill required by clinicians across the global medical landscape.^1^ The expectation to teach begins at the post-graduate level or residency level in most centres across North America and Europe.^2^ Most residency programs do have mandatory “residents as teachers” programming. However, the delivery of this curriculum is not standardized across centres.^2^^,^^3^   Furthermore, once residency training is complete, practicing clinicians may have challenges acquiring clinical teaching skills.^4^^,^^5^ A recent study of faculty members at various academic departments demonstrates that formal training for teaching physicians is perceived to be inadequate, with only 35% of those interviewed receiving any training focused on medical education skills.^6^

Recognizing that physicians start assuming educator roles upon entry into their residency programs, several institutions have offered formal teaching opportunities to senior medical students in preparation for this transition.^7^^,^^8^^,^^9^ To address the need for junior clinicians to develop insight into teaching skills and to the field of medical education in general, our institution developed a two-week elective entitled “An Introduction to the Art and Science of Clinical Teaching”.^10^ We intentionally situated the elective in the latter months of the fourth (and final) year of undergraduate medical school training. Most medical schools in North America follow the traditional Flexnerian model of medical education training, with the fourth year of medical school being one to consolidate core clinical knowledge and skills prior to residency.^11^ The inaugural year for our institutional elective was 2015. Since that time, 15–20 students per year have participated (this represents approximately 10% of the class). The curriculum was developed with input from key medical educators at our institution, and a student needs assessment survey.^11^^,^^12^ Areas of interest for students included enhancing clinical teaching skills, bedside teaching skills and developing mentoring skills. Of note, there was a lack of knowledge around scholarship in medical education; thus, the elective sought to address this gap as well.^12^  The elective is comprised of multiple components: a didactic portion consists of lectures from volunteer medical educators at our institution on various topics (e.g., the history of medical education, an introduction to major medical education theoretical frameworks, an introduction to assessment in medical education, bedside teaching, creating effective presentations, teaching in various settings (emergency room, ambulatory care)) and an introduction to creating effective journal club presentations.    A two-hour workshop is included to expose students to resources such as Med Ed Portal and perform a literature review in medical education. In prior offerings of the elective, practical opportunities were provided to assist in developing mentorship skills via an event entitled “Coffee with a Clerk”. Students were assigned to co-tutor physical examination skills for first- and second-year medical students. Participants are also provided asynchronous learning opportunities with online video presentations and readings to review. Finally, all students are required to deliver a 10-minute presentation on a topic of their choice using the presentation skills acquired during the elective. Students are evaluated on their level of engagement during the elective and the quality of their presentation.

During the COVID 19 pandemic, the elective was offered virtually. Most of the lectures and workshops were offered via the Microsoft Teams platform. Student presentations were delivered virtually as well. Due to social distancing requirements, practical junior peer teaching opportunities were not available. We were surprised to observe high levels of enrolment during the virtual offering of the elective, with no decline in participant numbers.

Previous studies have evaluated the perceptions and experiences of student participants of similar teaching electives offered at institutions across North America.^1^^,^^7^^,^^8^^,^^9^   These studies have noted high levels of student enjoyment, but challenges exist in ascertaining outcomes of the elective beyond student satisfaction. We sought to evaluate the experience of the elective of participating students via two focus groups (2017 and 2018 cohorts) and surveys. Ten students participated in the focus groups. Following the qualitative analysis of the focus group data, three concepts emerged regarding the utility of the elective – those of capacity, culture and community of practice building. There was a sense the elective fostered the acquisition of knowledge and skills regarding clinical teaching (in other words, capacity building in the field of medical education). This finding has been noted in other studies of medical students that engage in similar electives.^9^ Next, the medical students relayed the value of being exposed to professors and educators in the field of medical education (creating a culture of promotion of teaching).Finally, there was an appreciation of the need to form alliances with like-minded students who have an interest in becoming medical educators (building a community). Prior to the elective, students were acutely aware of the need to hone teaching-related skills and practices. At the conclusion of the elective, there was a recognition of the importance of the practical aspects of developing hands-on teaching skills and the continued need to obtain guidance on teaching skills during residency.

Future studies will be needed to examine the impact of the elective offered in the virtual format during the COVID 19 pandemic – and a shift in focus from observing and evaluating bedside and physical examination skills to evaluating virtual didactic presentations. Presently, 50% of medical schools in the United States (and only our institution in Canada) offer a dedicated fourth-year medical school elective in clinical teaching.^7^ Based on our experience and those of student participants at our institution, we would advocate for an expanded role for such programming to assist in preparing students to become future medical educators.

### Conflicts of Interest

The authors declare that they have no conflict of interest.
